# Plasma Lipids and Betaine Are Related in an Acute Coronary Syndrome Cohort

**DOI:** 10.1371/journal.pone.0021666

**Published:** 2011-07-01

**Authors:** Michael Lever, Peter M. George, Wendy Atkinson, Sarah L. Molyneux, Jane L. Elmslie, Sandy Slow, A. Mark Richards, Stephen T. Chambers

**Affiliations:** 1 Clinical Biochemistry Unit, Canterbury Health Laboratories, Christchurch, New Zealand; 2 Pathology Department, University of Otago, Christchurch School of Medicine and Health Sciences, Christchurch, New Zealand; 3 Department of Medicine, University of Otago, Christchurch School of Medicine and Health Sciences, Christchurch, New Zealand; Paris Institute of Technology for Life, Food and Environmental Sciences, France

## Abstract

**Background:**

Low plasma betaine has been associated with unfavorable plasma lipid profiles and cardiovascular risk. In some studies raised plasma betaine after supplementation is associated with elevations in plasma lipids. We aimed to measure the relationships between plasma and urine betaine and plasma lipids, and the effects of lipid-lowering drugs on these.

**Methodology:**

Fasting plasma samples were collected from 531 subjects (and urine samples from 415) 4 months after hospitalization for an acute coronary syndrome episode. In this cross-sectional study, plasma betaine and dimethylglycine concentrations and urine excretions were compared with plasma lipid concentrations. Subgroup comparisons were made for gender, with and without diabetes mellitus, and for drug treatment.

**Principal Findings:**

Plasma betaine negatively correlated with triglyceride (Spearman's *r_s_* = −0.22, p<0.0001) and non-high-density lipoprotein cholesterol (*r_s_* = −0.27, p<0.0001). Plasma betaine was a predictor of BMI (p<0.05) and plasma non-high-density lipoprotein cholesterol and triglyceride (p<0.001) independently of gender, age and the presence of diabetes. Using data grouped by plasma betaine decile, increasing plasma betaine was linearly related to decreases in BMI (p = 0.008) and plasma non-HDL cholesterol (p = 0.002). In a non-linear relationship betaine was negatively associated with elevated plasma triglycerides (p = 0.004) only for plasma betaine >45 µmol/L. Subjects taking statins had higher plasma betaine concentrations (p<0.001). Subjects treated with a fibrate had lower plasma betaine (p = 0.003) possibly caused by elevated urine betaine loss (p<0.001). The ratio of coenzyme Q to non-high-density lipoprotein cholesterol was higher in subjects with higher plasma betaine, and in subjects taking a statin.

**Conclusion:**

Low plasma betaine concentrations correlated with an unfavourable lipid profile. Betaine deficiency may be common in the study population. Controlled clinical trials of betaine supplementation should be conducted in appropriate populations to determine whether correction affects cardiovascular risk.

## Introduction

Betaine (trimethylglycine, “TMG”) is a dietary component [1], [2], and human betaine requirements can also be met by the metabolism of choline [3], [4]. Although it is a lipophobic metabolite, it has physiological interactions with lipids. These have been studied in farm animals and poultry because betaine is commonly included in feeds to produce leaner meat with a lower fat content [5], [6]. The effects of betaine on blood and tissue lipids have been documented in small animal models [7]–[9], suggesting that betaine supplementation has a favourable effect on obesity-related health risk factors. In a rat model, animals that gained more weight excreted more betaine than other animals on the same diet [10], and conversely, obesity in mice, induced by a high-fat diet, is associated with lowered liver betaine [11], raising the possibility that the lipid load can cause changes in tissue betaine. There is no consensus on how these observations relate to the known functions of betaine, which are as an osmolyte in most tissues, and as a store of methyl groups, mobilized in the liver by the action of betaine homocysteine methyltransferase [3],[4],[6]. There is conflicting evidence about the effect of betaine on lipids in human subjects. In normal subjects, betaine supplementation for short periods (6–12 weeks) may cause an increase in plasma betaine which appears to be associated with a small elevation in plasma low-density lipoprotein [12], [13], though this was not apparent after 6 months of supplementation [14]. In cross-sectional studies there is a negative relationship between plasma betaine and plasma lipids, particularly non-HDL cholesterol [15]. Cross-sectional results suggest that the negative relationship between betaine and blood lipids is most prominent in metabolic syndrome patients [15] and in those with lipid disorders [16], who may tend to be betaine deficient [3]. There is a gender difference in the associations, with the effects stronger in males than in females [15], [17].

We have examined an acute coronary syndrome cohort for relationships between betaine and its metabolite, *N,N*-dimethylglycine, with plasma lipids and with lipid-lowering drugs. Betaine is marketed as a nutritional supplement (often as “TMG”) for supposed cardiovascular benefits, although these claims are not supported by controlled clinical trials; if its effect is to raise plasma lipids there would be cause for concern. Conversely, cross-sectional results suggest that there is a subset of the population, possibly betaine-depleted [3], that may benefit from supplementation; if so, controlled trials with samples of those populations should be undertaken, and the target groups for this therapy defined [3], [4].

## Methods

### Subjects

This was a cross-sectional sub-study in an Acute Coronary Syndrome (ACS) cohort recruited by the Christchurch Cardioendocrine Group, Christchurch Hospital. Subjects had to have had ischaemic discomfort plus one or more of the following: ECG changes (ST segment depression or elevation of at least 0.5 mm, T-wave inversion of at least 3 mm in at least 3 leads, or left bundle branch block), elevated levels of cardiac markers, a history of coronary disease, or age of at least 65 years in patients with diabetes or vascular disease, i.e. identical criteria to those used in the OPUS-TIMI 16 trial and in De Lemos et al. [18], to be eligible for the study. Exclusion criteria: Severe co-morbidity limiting life expectancy to less than 3 years. Inability to provide written informed consent. For the betaine sub-study (**Table 1**) fasting plasma samples were collected on 531 subjects, and matching urine samples on 415 of these, at the four-month post-event follow-up clinic visit.

**Table 1:** Study population.

	Females	Males
Number	148	383
Median age (total range), years**	73 (51−91)	67 (55−93)
Number with diabetes	28	65
Number taking fibrates	5	7
Number taking statins*	118	336
Number taking ACE inhibitors	76	212
Number taking β-blockers	123	328
BMI (median, IQ range) kg/m^2^	27.0 (22.7−31.6)	26.5 (24.5−29.4)
Plasma triglyceride (median, IQ range) mmol/L	1.46 (1.05−1.96)	1.49 (1.03−2.06)
Plasma HDL-cholesterol (median, IQ range) mmol/L	1.25 (1.06−1.54)	1.07 (0.91−1.26)
Plasma non-HDL-cholesterol (median, IQ range) mmol/L	3.33 (2.53−4.31)	2.99 (2.50−3.67)
Plasma coenzyme Q (median, IQ range) µmol/L	0.65 (0.52−0.87)	0.70 (0.54−0.86)
Plasma betaine (median, IQ range) µmol/L***	38.9 (30.8−46.0)	44.5 (35.0−57.5)
Plasma DMG (median, IQ range) µmol/L*	3.4 (2.2−4.8)	3.8 (2.7−5.2)
Urine betaine/creatinine (median, IQ range) mmol/mole cr	8.2 (4.4−17.7)	9.2 (5.9−17.6)
Urine DMG/creatinine (median, IQ range) mmol/mole cr	2.4 (1.4−4.3)	2.9 (1.5−5.9)

cr: creatinine. DMG: *N,N*-dimethylglycine. IQ: interquartile. HDL: high density lipoprotein. Significance of gender differences: *p<0.05;

**p<0.01;

***p<0.001.

Study protocols were approved by the Canterbury Ethics Committee and all subjects gave written informed consent.

### Laboratory methods

Betaine and *N,N*-dimethylglycine were measured in plasma and urine by high performance liquid chromatography (HPLC) after separation of their 2-naphthacyl derivatives on Merck Aluspher alumina columns [19], [20]. Plasma total coenzyme Q_10_ was measured by HPLC with electrochemical detection [21]. Other biochemical measures in plasma and urine were all carried out using an Abbott ARCHITECT ci8200 Analyzer (Abbott Laboratories); plasma cholesterol was measured by an enzymatic cholesterol oxidase reaction, triglycerides by enzymatic hydrolysis of triglycerides, both using Abbott reagents and HDL cholesterol by an enzymatic reaction using Roche reagents (La Roche Ltd, Switzerland). Urine creatinine was measured by the Jaffé reaction.

### Statistical analysis

Statistical analyses were carried out using SigmaPlot for Windows version 11.2 (Systat Software Inc), which incorporates SigmaStat.

None of the data were normally distributed, even after log-transformation, therefore comparisons were based on ranking methods. Spearman's correlation coefficients were calculated. The populations were divided into tertiles of the plasma betaine or lipid fraction concentrations, and the differences of other metabolites in the tertiles were compared using the Mann-Whitney Rank Sum Test. Because of known gender differences in betaine metabolism [15], [17], male and female subgroups were analyzed separately. Diabetes is frequently associated with increased betaine excretion [3], therefore the effect of diabetes on the associations was assessed.

Multiple linear regression models were calculated to identify factors associated with plasma betaine and plasma lipid fractions. Initially, multiple best subset regression models (6 to 30) were calculated, including all plausible confounding factors, and the most consistently significant variables in these were combined in explicit multiple linear regression models. Where there was significant variance inflation both of the affected variables were added separately to explicit models. If some variables were no longer significant in these models, a new model was calculated omitting those variables, until models were obtained with all variables significant and with variance inflation factors (VIF) <2. Because of the smaller numbers of urine samples, separate best subset models were calculated including log-transformed urine excretions of betaine and *N,N*-dimethylglycine.

The data was divided into deciles of plasma betaine concentration to find underlying functional relationships between plasma betaine and lipid fractions. The median plasma betaine concentrations of the deciles were compared with the median BMI and plasma lipid concentrations of the same groups, using polynomial regression since not all relationships appeared to be linear.

## Results

### General associations between lipids and betaine

In the total study population there were small but significant correlations between betaine, its metabolite, *N,N*-dimethylglycine, and plasma lipid fractions (**Table 2**). Higher plasma betaine concentrations was associated with lower triglyceride and non-HDL cholesterol concentrations (and also with lower calculated LDL cholesterol). Coenzyme Q circulates mainly in low-density lipoproteins, so it is not surprising that it also negatively correlated with betaine; however, the ratio of coenzyme Q to non-HDL cholesterol (or to calculated LDL-cholesterol) positively correlated with betaine. The correlation between HDL cholesterol and the ratio of dimethylglycine to betaine is *r_s_* = –0.15 (p = 0.0005).

**Table 2:** Correlations in all subjects1.

	Plasma betaine	Plasma DMG^2^	Urine bet/creat^3^	Urine DMG/cr^3^
Triglyceride	**−0.220 (<0.0001)**	+0.026 (0.55)	+0.04 (0.41)	–0.026 (0.60)
HDL-cholesterol	+0.023 (0.61)	**−0.130 (0.0031)**	–0.004 (0.94)	–0.05 (0.28)
Non-HDL-chol^4^	**−0.269 (<0.0001)**	–0.077 (0.079)	–0.007 (0.89)	–0.05 (0.34)
CoenzymeQ_10_	**−0.129 (0.0033)**	–0.04 (0.37)	**+0.098 (0.048)**	**+0.111 (0.025)**
CoQ/Non-HDL-c	**+0.134 (0.0024)**	+0.005 (0.92)	**+0.165 (0.001)**	**+0.193 (0.0001)**
BMI	**−0.158 (0.00047)**	–0.003 (0.94)	–0.04 (0.39)	–0.026 (0.62)
Age	+0.054 (0.22)	**+0.109 (0.013)**	+0.038 (0.43)	+0.028 (0.58)

1 Spearman's correlation coefficients (with p values). Significant (p<0.05) correlations indicated in bold.

2 DMG: Dimethylglycine.

3 Excretions expressed as ratio of urine betaine or DMG to creatinine.

4 Non high density lipoprotein cholesterol.

**Figure 1:**
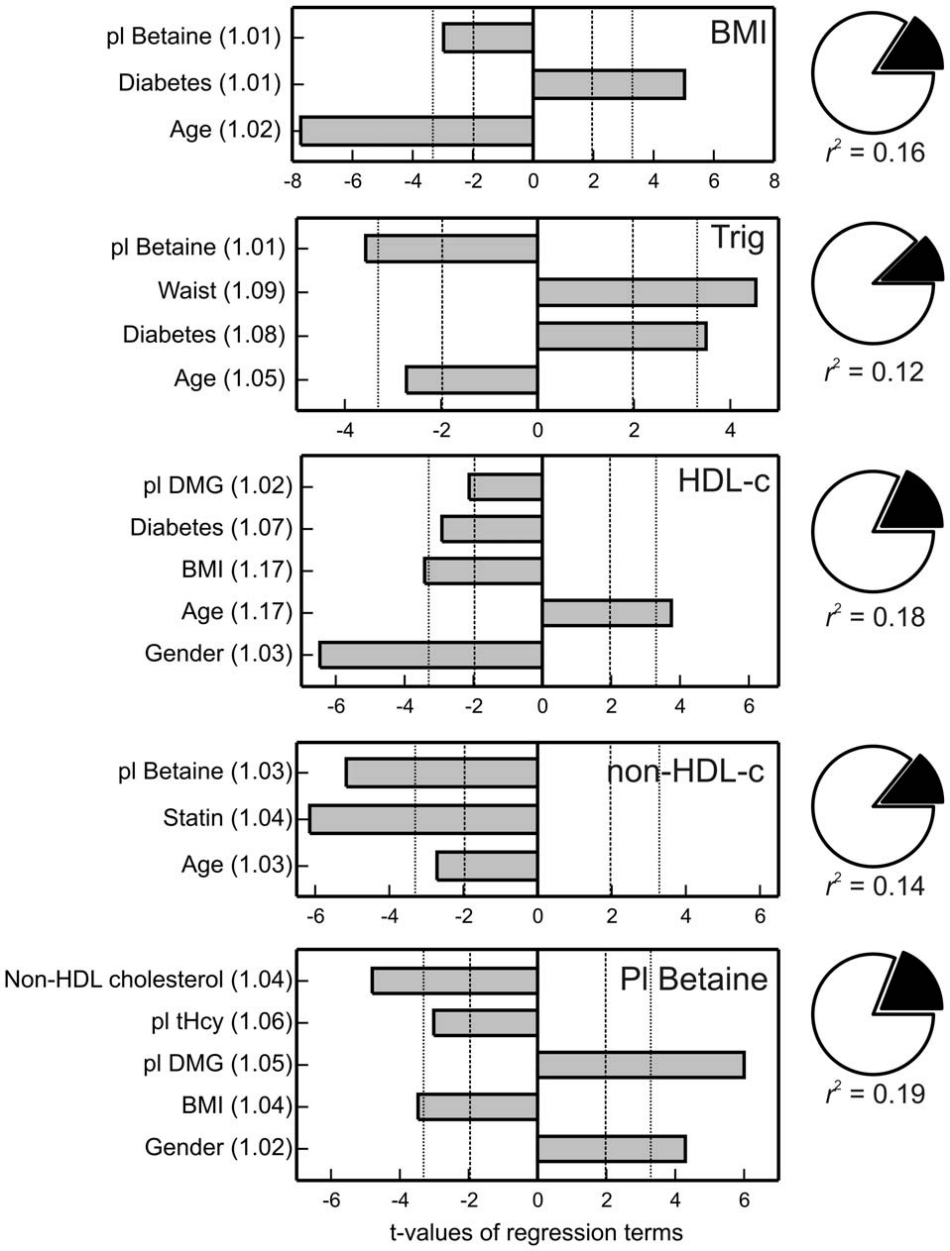
Regression variables affecting plasma betaine concentrations, plasma lipid fractions and body mass index (BMI). Trig: plasma triglyceride concentration; HDL-c: HDL cholesterol; non-HDL-c: non-HDL cholesterol (total cholesterol - HDL cholesterol). The bars are the t-values of the significant variables, negative for variables with negative coefficients and positive for those with positive coefficients. Dashed vertical lines indicate p = 0.05; dotted vertical lines p = 0.001. Numbers in brackets after variable names are the variance inflation factors. Pie figures on the right show the portion of variance explained by regression (black). DMG: dimethylglycine.

Best subset regression models (30) were calculated to identify factors that appear to predict the plasma betaine concentrations. None of these explained more than 25% of the variance in plasma betaine (*r* ^2^<0.25), and notable factors that did not appear as significant in any of them included age, diabetes, and plasma glucose, electrolytes, triglyceride and creatinine. Using consistently significant variables, explicit multiple regression models (**Figure 1**) were calculated that showed that non-HDL cholesterol was highly significantly negatively influenced by plasma betaine concentrations and this was independent (VIF 1.04) of other significant factors; these included BMI (negative, VIF 1.04) and gender (VIF 1.02). Plasma triglyceride only appeared as a significant factor if non-HDL cholesterol was excluded from the model. Conversely, factors significantly affecting plasma betaine concentrations included and (negatively) BMI and non-HDL cholesterol concentrations (**Figure 1**), independently of other factors such as age, diabetes and statin treatment. Plasma lipids were not included in these models because of multicollinearity.

Plasma betaine concentrations appeared to be positively associated with HDL-cholesterol in all 6 best subset models but ceased to be significant when BMI was included in an explicit multiple regression model rather than waist (only one of waist or BMI was included to eliminate multicollinearity). The model for HDL-cholesterol shown in **Figure 1** can be slightly improved by using the ratio of plasma dimethylglycine to betaine instead of plasma dimethylglycine concentrations; this ratio term is more significant (t = –3.13, p = 0.002) than the concentration. Variables that did not enter any of the best subset models as significant factors include plasma homocysteine, creatinine and urea. Age was not a significant factor in any of the models for plasma betaine. The urinary excretions of betaine and dimethylglycine were not significant in any of the models for predicting plasma lipids or BMI that were calculated that included these urinary measures.

In none of the models in **Figure 1** was the explained variance more than 20% of the total variance (*r* ^2^<0.2).

Blood pressure was not recorded in this study. Clinically-diagnosed hypertension was recorded as a variable (0 or 1) but given that the majority of subjects were receiving medication that would modify blood pressure (**Table 1**) this was regarded as a compromised measure; it did not enter into any of the best subset models for blood lipid fractions, though it was a significant predictor of BMI.

**Figure 2:**
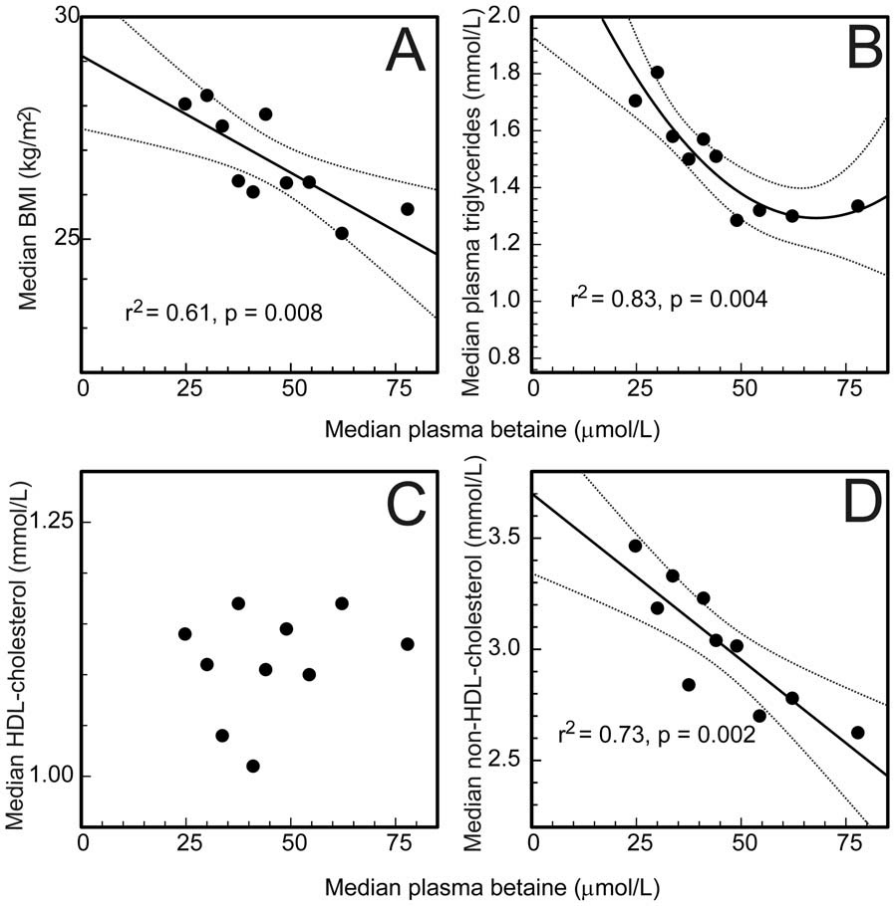
Plasma lipids and BMI as a function of plasma betaine concentrations. Data grouped into deciles based on the plasma betaine concentrations, and median betaine concentrations of these plotted against A: median BMI, and the medians of the plasma lipid fractions (B: triglycerides, C: HDL cholesterol, D: non-HDL cholesterol). Regression lines (with 95% confidence intervals) shown when significant, straight lines (A&D) or curved (B) where the second order term is significant (p = 0.035).

BMI and plasma non-HDL cholesterol concentrations decreased approximately linearly with increasing plasma betaine concentrations (**Figure 2**), but the relationship with plasma triglyceride concentrations was not linear, the binomial term (**Figure 2B**) being significant (p = 0.035). Above a plasma betaine concentration of about 45 µmol/L no association was detected between plasma betaine and triglyceride concentrations, whereas low plasma betaine concentrations were strongly associated with elevated plasma triglycerides. No significant relationship was detected between plasma betaine and plasma HDL-cholesterol (**Figure 2C**).

### Comparisons in genders

**Figure 3:**
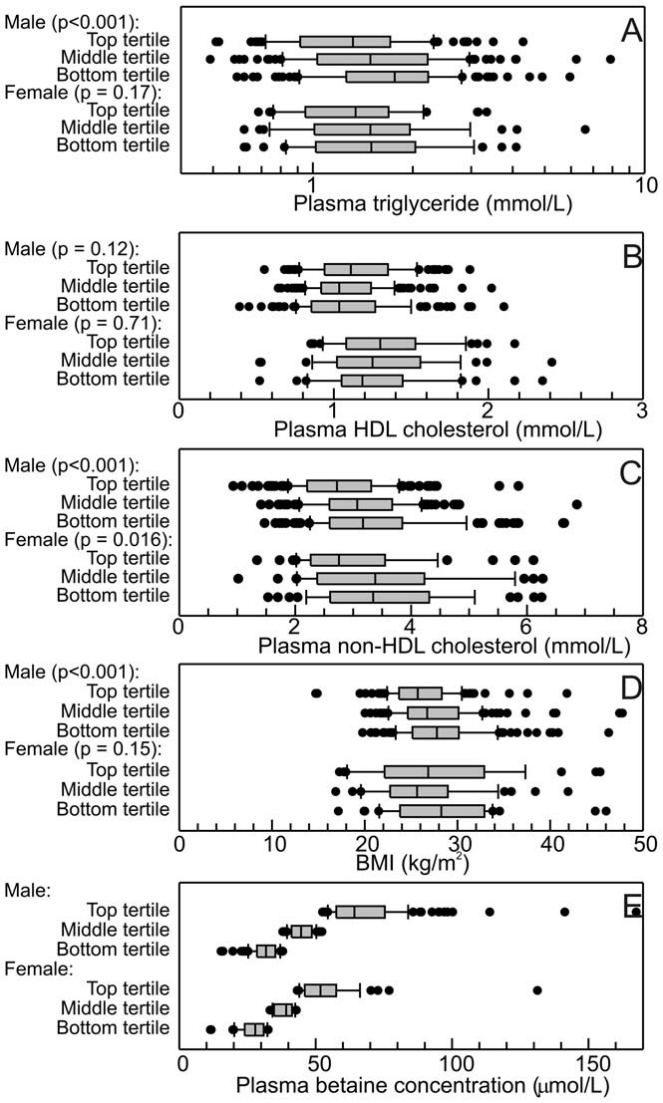
Plasma lipids and BMI in male (*n* = 382) and female (*n* = 150) patients with different tertiles of plasma betaine concentrations. The solid boxes show the interquartile ranges, with the medians indicated, and the cap the 90%-ile, of the (A–C) plasma lipid concentrations in the subject groups, D the BMIs of the subjects, and E. for comparison, the plasma betaine concentrations in the tertiles. The significance (left) indicates whether there was a difference between the tertiles (ANOVA on ranks).

The negative correlations between plasma betaine and triglyceride or non-HDL cholesterol were highly significant in the male subjects (Spearman's *r_s_* = –0.25, p<0.0001 for triglyceride, *r_s_* = –0.26, p<0.0001 for non-HDL cholesterol). They appeared to be weaker in female subjects (*r_s_* = –0.16, p = 0.051 for triglyceride, *r_s_* = –0.22, p = 0.007 for non-HDL cholesterol) though the differences between the correlation coefficients were not significant. In males, top tertile median of plasma betaine concentrations was 20 µmol/L (44%) higher than in the middle tertile, whereas the lower tertile plasma betaine concentrations was about 13 µmol/L (29%) lower. These differences tended to be associated with lower plasma triglyceride, lower non-HDL cholesterol or low BMI, and possibly with higher HDL-cholesterol (**Figure 3**). In the smaller cohort of female patients the only significant trend was with non-HDL cholesterol. The difference in median plasma betaine concentration between top and bottom tertiles was associated with approximately 25% lower plasma triglyceride and 14% lower non-HDL cholesterol (**Figure 3**). The relationship between plasma betaine and total coenzyme Q was dominated by the strong relationships each had with non-HDL cholesterol, giving a negative correlation, but the ratio of coenzyme Q to non-HDL cholesterol (a measure of CoQ saturation of lipoproteins) was positively associated with plasma betaine, at least in male patients.

The converse relationships were also significant. In males, subjects in the bottom tertile of plasma triglyceride or bottom tertile of plasma non-HDL cholesterol had significantly higher plasma betaine concentrations (p<0.001) and those in the bottom tertile of HDL-cholesterol or coenzyme Q_10_/non-HDL cholesterol had lower plasma betaine (p = 0.022 for HDL-cholesterol, p = 0.002 for the CoQ ratio). In females the apparent trends were the same but were only statistically significant for non-HDL cholesterol (p = 0.001).

Plasma dimethylglycine did not correlate with either triglyceride or with non-HDL cholesterol in either gender, but surprisingly there was a negative correlation (*r_s_* = −0.19, p = 0.023) between plasma dimethylglycine concentrations and HDL cholesterol in females (in males, the correlation was not significant, *r_s_* = −0.09, p = 0.095). When the ratio of dimethylglycine to betaine, an indicator of active betaine metabolism, was compared with the HDL cholesterol concentrations, there was a significant negative correlation in both genders (*r_s_* = −0.15, p = 0.0048 in males; *r_s_* = −0.24, p = 0.0047 in females).

Neither the urinary excretion of betaine nor of dimethylglycine correlated with any of the plasma lipid fractions, in either gender. The positive correlations of these urinary excretions with the coenzyme Q to non-HDL cholesterol, or to calculated LDL-cholesterol (**Table 2**) were not significant in the female subjects, and were weak or not significant in the male subjects (p = 0.092 for betaine excretion, p = 0.023 for dimethylglycine excretion) suggesting that the results in Table 2 were strongly influenced by gender differences in the variables being compared.

### Effects of diabetes

As expected, diabetes is a significant factor in determining BMI and plasma lipids (**Figure 3**) but not plasma betaine. Its contribution to the multiple regression models is independent of that of plasma betaine. In the groups of male and female patients without diabetes, the same significant relationships were seen as those in the whole male and female samples. The relationships between plasma betaine and lipids were not detected as statistically significant in the small groups of patients with diabetes (n = 92). The apparent trends were not significantly different from the relationships seen in the total cohort, but there was insufficient power to confirm that these trends were significant in the subgroup. Only 4 subjects were diagnosed with type 1 diabetes.

Diabetic subjects frequently excrete excessive betaine [3], and in this subgroup betaine excretion positively correlated with non-HDL cholesterol (*r_s_* = +0.25, p = 0.044, n = 67). This association was not seen.in subjects without diabetes (*r_s_* = −0.06, p = 0.32, n = 329).

### Effect of lipid-modifying drugs

**Figure 4:**
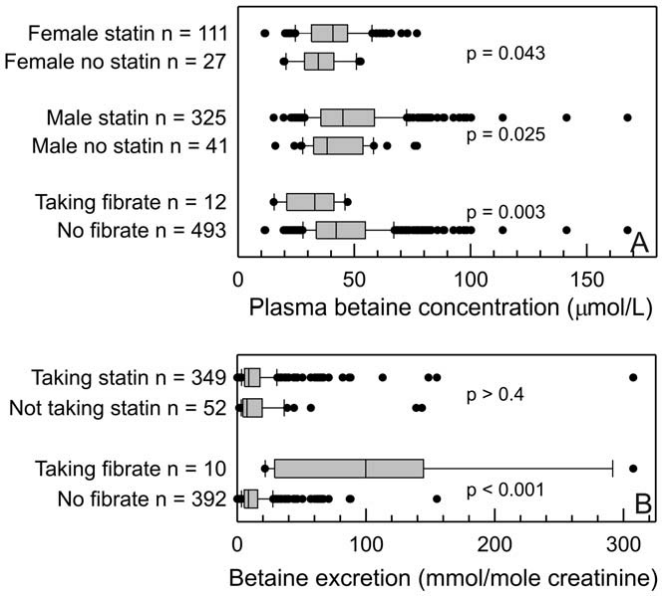
Plasma betaine concentrations in subjects being treated with lipid-lowering drugs; A: plasma betaine concentrations and B. urinary betaine excretions. The solid boxes show the interquartile ranges, with the medians indicated, and the cap the 90%-ile, of the A. plasma betaine concentrations and B. urinary betaine excretions in the subject groups. The significance of the differences (Mann-Whitney Rank Sum Test) are shown.

The majority of the subjects were being treated with statins (**Table 1**), and overall the median plasma betaine concentration in the statin-treated patients (43.0 µmol/L) was higher than in those not receiving statins (36.5 µmol/L, p<0.001). This was true for both genders (**Figure 4**). There was no detectable difference in the betaine excretions between subjects receiving statins and those not (**Figure 4**). The median plasma dimethylglycine (3.7 µmol/L) was the same in both groups, as was the dimethylglycine excretion (2.7 mmol/mole creatinine). The subjects not receiving statins tended to be slightly older. There were only modest differences in the lipid profiles between the subjects receiving or not receiving statins, as expected the plasma cholesterol in the subjects on statins (median 4.2 mmol/L) was lower than in those not (5.2 mmol/L), but the triglyceride and HDL-cholesterol concentrations were almost identical. Total coenzyme Q concentrations were also slightly lower (median 0.67 µmol/L *cf* 0.74 µmol/L, p = 0.004) in the subjects (both sexes) treated with statins, though the ratio of coenzyme Q to non-HDL cholesterol was higher in the treated patients (median 0.227 *cf* 0.195, p<0.001); a similar difference was seen when the ratio of coenzyme Q to LDL cholesterol was calculated.

**Figure 5:**
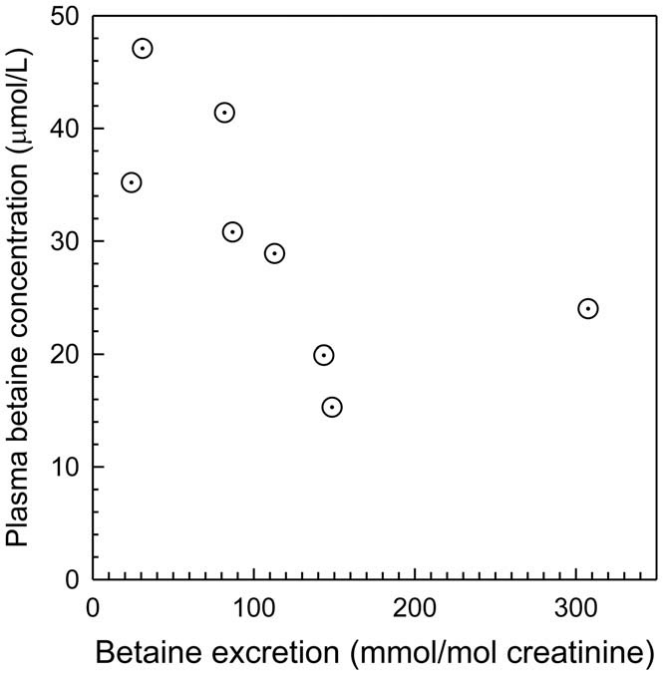
Plasma betaine concentration as a function of urine betaine excretions in subjects treated with fibrate. Spearman's r = −0.86; p = 0.0018.

There was plasma betaine data on only 12 subjects who were being treated with a fibrate, all with bezafibrate. The median plasma betaine concentration in these was lower than in subjects not treated with fibrate (**Figure 4**). The urine betaine excretion was estimated on 10 subjects treated with fibrate, and the median excretion was significantly higher (p<0.001) than in those not receiving fibrate (**Figure 4**). The urine dimethylglycine excretion (median 8.8 mmol/mole creatinine) was also significantly (p<0.001) higher than in subjects not taking fibrates (2.6 mmol/mole creatinine). The plasma betaine concentration and betaine excretion were negatively correlated in the fibrate-treated group (Spearman's *r_s_* = −0.86; p = 0.0018), significant despite there being matching data on only 8 subjects. This suggested that betaine loss may be an important factor in the lower plasma betaine found in these subjects (**Figure 5**), and this association was not apparent in other subgroups. The subjects treated with bezafibrate had a more abnormal lipid profile than other subjects. The median plasma triglyceride of 1.75 mmol/L was not significantly higher than the 1.48 mmol/L for non-fibrate treated subjects, but the median non-HDL cholesterol of 3.83 mmol/L was significantly higher than in subjects not taking a fibrate (3.07 mmol/L, p = 0.002).

The estimates of the associations of betaine and plasma lipids were not significantly changed by excluding the subjects taking fibrates.

Only four subjects in this study were taking nicotinic acid. The median plasma betaine concentration in the treated subjects was 47.0 µmol/L, not significantly different from the median in the other 515 subjects (42.0 µmol/L). In contrast to the fibrate group, the median urine betaine excretion in the nicotinic acid treated group (n = 3) was 3.3 mmol/mole creatinine compared with 9.1 mmol/mole creatinine in the other subjects (n = 414; p = 0.092). While the numbers are too small to draw conclusions, there was no suggestion of an increase in excretion comparable to that seen with fibrate treatment; if anything it was more likely to be in the reverse direction.

## Discussion

### Interaction of plasma betaine and lipids

Plasma betaine and lipids clearly interact, with 15–20% of the variance in each being associated with variance the other. Although the causal direction has not been assumed here, it is more likely that betaine is affecting the lipids. A reason to add this assumption is the evidence that an increased betaine intake increases plasma betaine and favorably affects lipid distribution in the body; the animal industries include betaine in animal feeds to produce better meat, more lean muscle mass and less fat [5], [6], in other words, to decrease obesity. These effects are best documented in pigs, both in growing piglets and in adult animals, but similar effects are well known in poultry [22], [23]. In small animal models, when hyperhomocysteinemic mice were supplemented for a year, plasma triglycerides decreased while HDL-cholesterol increased; other improvements in the atherogenic risk factor profile were also observed [8]. Betaine therapy also attenuates atherosclerosis in the apoE knockout mouse [9]. However, human intervention studies have suggested an opposite effect on plasma lipids: healthy but overweight subjects supplied a betaine supplement to increase their plasma betaine concentrations had a significant increase in plasma cholesterol [12], and Olthof et al. [13] questioned the safety of betaine supplementation on the basis of a small elevation in plasma triglycerides and LDL cholesterol in subjects receiving betaine supplements. Betaine supplementation can increase plasma lipids in small animal models; in an apolipoprotein E-deficient mouse model, which develops atherosclerotic lesions [9], betaine supplementation increased plasma cholesterol (though not triglycerides) at the same time as it decreased atherosclerotic lesion areas. The physiological significance of the possible rise in plasma lipids during betaine supplementation has been questioned [24], and a group of non-obese healthy younger adults treated with betaine for six months showed insignificant changes in plasma lipids [14]. Another study detected a small decrease in plasma lipids after a modest betaine supplementation [25].

### Resolving the contrary indications from cross sectional studies

A large cross-sectional study of a Norwegian population [15] showed a highly significant negative association between plasma betaine concentrations and both plasma lipids and markers of obesity (BMI, percent body fat and waist circumference). Consistent with the results reported here, higher plasma betaine was associated with plasma triglyceride and non-HDL cholesterol, with the effect more pronounced in men than in women; there was a trend for HDL cholesterol to be weakly positively associated with plasma betaine but this was only statistically significant in younger (47–49 years) males. These results are consistent with the suggestion that betaine can be deficient in patients with symptoms of the metabolic syndrome [3], and because of the tight regulation of plasma betaine [26], [27] this may not always be detected as plasma concentrations do not reliably reflect tissue levels. Here, we have shown that the association of betaine with BMI and lipid components of the metabolic syndrome are clearly independent of each other and of potential confounders such as gender, age and diabetes (variance inflation factors <1.05 in multiple regression models).

Other animal studies suggest that betaine affects the partitioning of lipid in the body, and in particular increases the export of tissue triglycerides. In normal rats, betaine supplementation (especially when the methionine supply is restricted) leads to increased hepatic production of apolipoprotein B [28] and increased plasma LDL and triglycerides, and these changes are associated with a large decrease in tissue lipid, which has been confirmed in other studies [7]. The overall effect of increased betaine intake probably depends on other dietary components (as shown in rats [7]), the presence of disease, and tissue lipids. Two contrasting studies in pigs illustrate this point: betaine supplementation increased plasma lipids in castrated pigs of a genetically obese strain [29], whereas a lower level of betaine supplementation decreased plasma lipids in non-obese female pigs [30]. A possible dose dependence of the effect of plasma betaine on lipids was suggested by Konstantinova et al. [15], and it is also plausible that the effect of betaine on plasma lipids depends on tissue lipid stores. This is consistent with cross-sectional human data which suggests that those subjects with the combination of elevated triglyceride and non-HDL cholesterol with low plasma betaine are likely to show features of the metabolic syndrome [15], [16].

In a contrasting recent cross-sectional study, plasma concentrations of trimethylamine *N*-oxide, choline and betaine are all reported to be associated with an increased number of vascular events in an at-risk population [31]. It is proposed that the causal agent is trimethylamine or its *N*-oxide; trimethylamine is produced in the gut by bacterial metabolism of choline and oxidized in the liver to its *N*-oxide. This cannot account for the betaine results because betaine is not converted to trimethylamine in the gut [32]. Betaine concentrations in tissues, where it functions as an osmolyte, are orders of magnitude higher than in plasma [33] therefore elevated betaine concentrations themselves are unlikely to cause damage. Our preliminary prospective results (unpublished data) could reconcile these inconsistencies: although low plasma betaine concentrations were a more significant risk factor for myocardial infarction, there was also an increased risk associated with the highest concentrations, and we speculate that there were some patients whose control of betaine efflux from tissues is compromised, and this subgroup is prone to early events.

### Cross-sectional studies and mechanisms

Cross-sectional studies do not demonstrate causality, but generate hypotheses. This is also illustrated by the effects of lipid lowering drugs on plasma betaine concentrations and urinary betaine excretion. Are the differences caused by the drug treatment, or do they reflect differences in the patients that are prescribed those drugs? In the case of statins, there is no reason to suspect that the patients treated with statins differed significantly from the minority who were not receiving statins, and it is likely that an increase in plasma betaine is another pleotropic effect of statins[34]. Fibrates, however, almost certainly cause increased betaine excretion [35] and since this is negatively correlated with the plasma betaine it is plausible to suggest that it is a cause of the lower plasma betaine in these patients. Fibrate treatment is therefore inducing a betaine deficiency, which may compromise the benefits of fibrate. Subjects being prescribed fibrate would be expected to have elevated triglyceride and low-density lipoprotein, so finding a difference in these (and the LDL- associated coenzyme Q) in fibrate-treated patients is a trivial result that has no necessary connection with the differences in plasma betaine. There were too few subjects taking nicotinic acid to detect any significant effects on plasma and urine betaine, but the limited data suggests that this drug does not cause a comparable effect to that seen with bezafibrate. The negative effect of statin therapy on plasma coenzyme Q is well-known, but when expressed as a ratio to non-HDL cholesterol (or LDL-cholesterol) the apparently available coenzyme Q is higher in patients receiving statins. There is a similar association between plasma betaine and coenzyme Q, suggesting that higher plasma betaine concentrations are associated with a more favorable coenzyme Q status.

Konstantinova et al [15] concluded that metabolic syndrome patients had lowered plasma betaine concentrations but elevated plasma choline concentrations. This is consistent with a conjecture that betaine deficiency is not generally associated with choline deficiency, but instead betaine deficiency may often reflect impaired mitochondrial conversion of choline to betaine, a major source of betaine. Choline is itself essential and central to phospholipid (and hence lipoprotein) synthesis, and betaine is not a source of choline. The effects of each on lipid metabolism seem to be independent of the other, but again this is surmised from cross-sectional data and needs to be verified experimentally. Vascular disease can cause an increase in plasma free choline [36] (as a result of phospholipid breakdown), which complicates interpretation of the relationship between plasma choline and betaine: plasma betaine is normally controlled by the enzyme betaine homocysteine methyltransferase in the liver [3], [37]. This explains why, in several studies [15], [31], the positive correlations between plasma choline and betaine are modest (typically *r*∼0.3). Our study supports the earlier report [15] that the metabolic syndrome is often associated with a combination of low plasma betaine and an atherogenic lipid profile by showing that BMI and plasma lipids are independently related to plasma betaine. Although this cross-sectional data does not demonstrate causality, the relationship between plasma triglycerides and betaine is consistent with the suggestion of Konstantinova et al. [15] that the effects of betaine are dose dependent, so that increasing the betaine intake of a betaine replete subject may not necessarily be beneficial.

Our, and other, cross-sectional results suggest a testable hypothesis that a betaine deficiency is associated with an atherogenic lipid profile. Further hypotheses are that betaine supplementation of betaine-deficient subjects will improve their lipid profiles, and will decrease their vascular risk, though there might be a short term increase in plasma lipids in obese subjects. These hypotheses suggest that long-term controlled trials of betaine supplementation are needed, especially ones that target populations that are likely to be betaine deficient. Previous human supplementation studies have excluded subjects likely to be betaine deficient [12], [14]. In the present study, the difference in plasma betaine between top and bottom tertiles is about 30 µmol/L, and this is associated with an approximately 25% difference in plasma triglyceride and a 14% difference in non-HDL cholesterol. These data imply substantial differences in cardiovascular risk. It should be tested whether changing the betaine concentrations alters risk: increasing plasma betaine by regular supplementation is easily achieved and inexpensive. Hypotheses about mechanisms need to consider both the osmolyte role of betaine, and (especially in view of the relationship between dimethylglycine and HDL-cholesterol) the methyl store role of betaine, and may best be addressed in animal models where tissue betaine contents can also be measured.

### Limitations

As discussed, the cross-sectional design only generates hypotheses and does not demonstrate causality. The limited numbers in subgroups (especially female subjects and subjects with diabetes) means that there are some negative results that should be regarded with caution. The population is a selected one (with established vascular disease and highly medicated) and therefore the results of this study do not necessarily reflect those that would be found in the general population.
